# Impacts of dredging and restoration on sedimentary carbon stocks in seagrass meadows of Pari Island, Indonesia

**DOI:** 10.1038/s41598-025-03870-3

**Published:** 2025-07-15

**Authors:** Yusmiana P. Rahayu, Gary A. Kendrick, Pere Masqué, Wawan Kiswara, Hadiwijaya L. Salim, Ali A. Lubis, Mathew A. Vanderklift

**Affiliations:** 1 School of Biological Sciences and Oceans Institute, The University of Western Australia, 35 Stirling Highway, Crawley, WA 6009 Australia; 2 Research Center for Conservation of Marine and Inland Water Resources, National Research and Innovation Agency, The Republic of Indonesia, Soekarno Science and Technology Area, Jl. Raya Bogor Km 46, Cibinong, Bogor, 16911 Indonesia; 3 School of Natural Sciences, Center for Marine Ecosystems Research, Edith Cowan University, Joondalup, WA 6027 Australia; 4 Yayasan Lamun Indonesia (LAMINA), Jl. Amonia No. F10, Kavling Pupuk Kujang, Beji Timur, Depok, 16422 Indonesia; 5 Research Center for Radiation Process Technology, National Research and Innovation Agency, The Republic of Indonesia, BJ. Habibie Area, Jl. Puspiptek, Muncul, Banten 15314 Indonesia; 6 CSIRO Environment, Indian Ocean Marine Research Centre, Crawley, WA 6009 Australia

**Keywords:** Blue carbon, Nature-based solutions, Climate change mitigation, Carbon credit, Conservation, Carbon cycle, Biogeochemistry, Climate sciences, Environmental sciences, Ocean sciences

## Abstract

The effects of dredging and restoration on carbon sequestration in seagrass sediments is not well understood. Our knowledge is derived from few studies conducted in areas affected by dredging or restoration, the majority of which are from temperate regions. There is limited information available for tropical regions, where seagrass sediments can differ greatly in species composition, geomorphology, and hydrodynamic conditions. This study aims to assess the effects of dredging and seagrass restoration on sediment organic carbon concentrations and stocks at Pari Island, Indonesia. The results indicated that sediment organic carbon concentrations (%C_org_) were higher in persistent seagrass meadows than in dredged, restored seagrass, and bare sand areas. Dredging at Pantai Perawan, Pari Island, led to the removal of seagrass vegetations and sediments. We found that the organic carbon concentrations in the sediment of dredged areas were 17% lower than that of persistent seagrass sediments. Although seagrass restoration has increased seagrass cover, the organic carbon concentrations in restored meadows were still lower than in persistent meadows. Therefore, we inferred that there have been no significant gains in organic carbon concentrations or stocks in sediments under the restored seagrass after 13 years. Protecting existing seagrass meadows is fundamental because restoring seagrass after disturbance and re-establishing carbon sequestration requires considerable time and resources.

**Supplementary Information:**

The online version contains supplementary material available at 10.1038/s41598-025-03870-3.

## Introduction

Seagrasses are underwater plants found in shallow coastal waters. Seagrass ecosystems provide food and shelter for many species and multiple benefits to humans, including protecting shorelines from erosion^1–3^. Together with mangroves and tidal marshes, they are commonly referred to as ‘blue carbon’ ecosystems because of their ability to sequester organic carbon in their biomass and underlying sediment^4–6^. Protection and restoration of blue carbon ecosystems are potentially effective nature-based solutions for climate change mitigation^5,7^.

Seagrasses use carbon dioxide and dissolved inorganic carbon during photosynthesis^8^. Some of the organic carbon produced from photosynthesis is stored in seagrass biomass, and seagrasses also entrain particles of autochthonous and allochthonous origin into the sediment in which they grow so that in some conditions it can accumulate a large amount of organic carbon^9,10^. Slow oxygen movement through water, combined with anoxia in the sediment slows decomposition^4^, creating an environment which is conducive to storing carbon for a long time, if protected from anthropogenic disturbances.

However, the increasing severity of weather events like marine heatwaves and cyclones, and human activities like dredging and wastewater discharge pose a significant challenge to the persistence of seagrass ecosystems^11–16^. Activities such as dredging sometimes involve excavation of seagrass meadows, leading to complete loss or fragmentation^17^. Marine heatwaves and high nutrient concentrations cause physiological stresses to seagrass that can lead to widespread mortality^18–20^. In both cases, the organic carbon sequestered in the sediments below the seagrass meadows can be remineralised and released as greenhouse gases^21–23^.

To date, the rate and extent to which dredging and restoration influence the carbon sequestration capacity of seagrass sediments remain uncertain. Our understanding has been gleaned from only a few studies conducted at locations where dredging or restoration has occurred, most of which are in temperate regions. For example, Thorhaug et al*.*^16^ studied the loss of organic carbon due to various types of disturbances, including dredging, and the gain from seagrass restoration in the Gulf of Mexico. Their results suggested a return of organic carbon in the sediment soon after restoration (< 3 years). However, other studies indicate that it may take longer to observe such changes. For instance, carbon sequestration rates returned to levels comparable with persistent meadows within 18 years in Oyster Harbour, Australia^24^, and 12 years after seagrass restoration in Virginia, USA^25^. Limited data exist for tropical regions, where the nature of seagrass sediments often differs significantly in terms of species composition, geomorphology and hydrodynamic conditions.

**Fig. 1:**
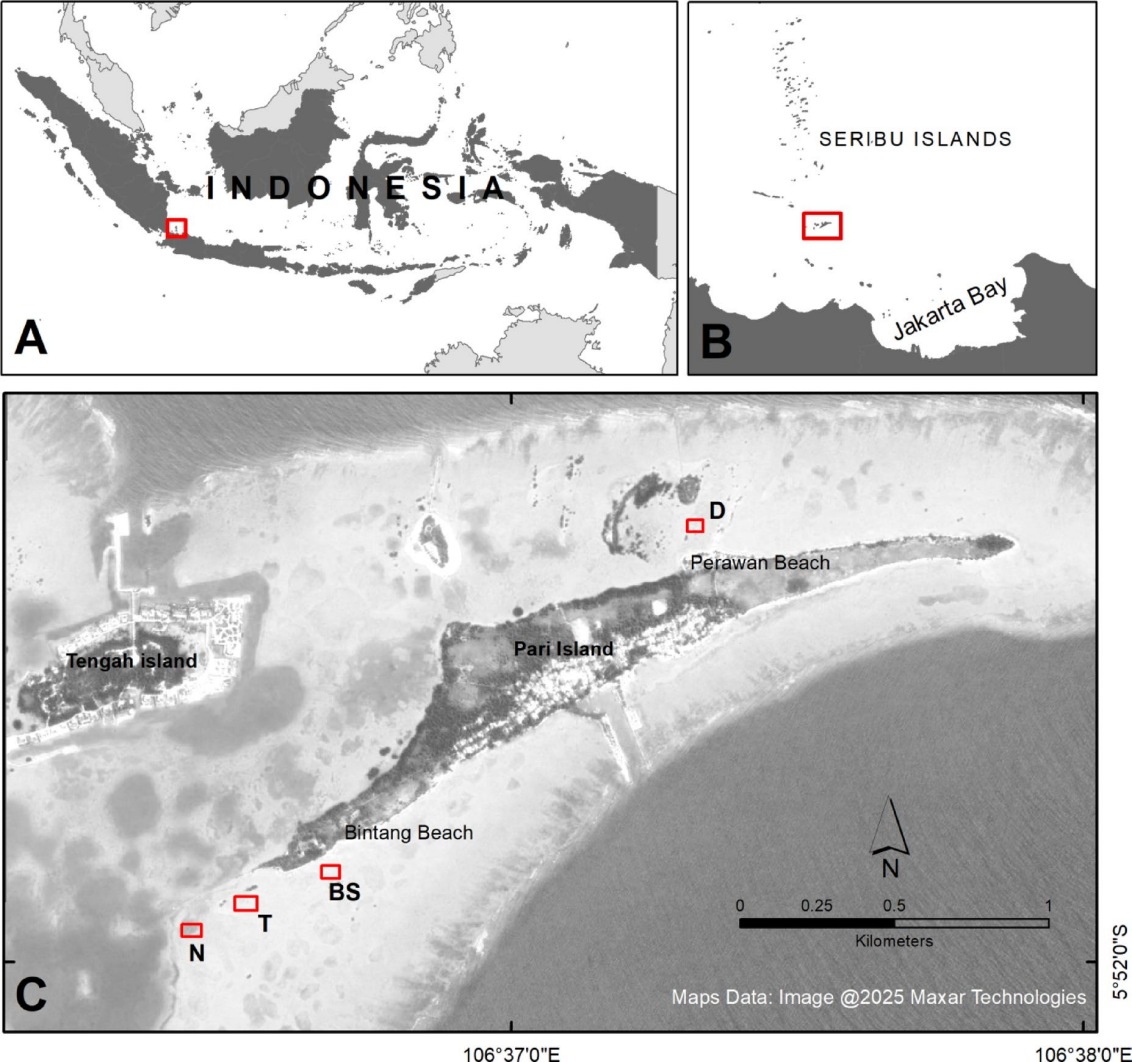
Location of Pari Island and study sites. (**A**) Map of Indonesia with Pari Island (red box). B. Pari Island (red box) in Seribu Islands. C. Survey sites are indicated by red boxes, with N = persistent seagrass, T = restored seagrass, BS = bare sand, D = dredged areas. Maps were created using QGIS version 3.22.14 (Białowieża, https://qgis.org).

Pari Island is a small island in Seribu Islands (off the northern coast of Java, Indonesia) with coral reef, mangrove and seagrass ecosystems^26^ (Fig. 1). Pari Island has experienced coastal flooding and erosion^27,28^ as well as decreasing groundwater quality^29^. Between 2010 and 2016, it experienced increasing tourism and coastal development^30^ that led to dredging adjacent to a popular tourist beach on the northern side of Pari Island (Pantai Pasir Perawan) to facilitate swimming in 2017. Local authorities and communities in Seribu Islands built concrete seawalls, breakwaters, and conducted mangrove planting in 2004 at several locations, including Pari Island^28^. On the western side of the island, experimental seagrass restoration using transplants was initiated in 2009^31^.

In this study, we aim to assess the impact of dredging and seagrass restoration on sediment organic carbon stocks and accumulation rates at Pari Island, Indonesia. We predicted that C_org_ concentrations and stocks in the sediment of persistent seagrass meadows would be higher than that in dredged areas, restored areas, and in unvegetated areas where seagrasses do not occur naturally. We also predicted that the C_org_ concentrations and *δ*^13^C of organic carbon (an indicator of carbon sources) in restored areas would resemble those in the sediment of persistent meadows.

## Methods

### Study area

The study was conducted at Pari Island, located within the Seribu Islands archipelago, part of the DKI Jakarta Province in Indonesia (Fig. 1). The Seribu Islands consist of 114 islands, ranging from 0.04 to 59.9 hectares in size. No rivers or springs are present on the Seribu Islands, including Pari Island^28^. Pari Island has a fringing reef^26,32^ and spans approximately 41 hectares. Fishing and tourism are the primary livelihoods of its inhabitants^30,33^. The island’s population increased from approximately 400 in 1995^34^ to 3000 individuals in 2021^33^. Pari Island is a prominent tourist destination due to its proximity to Jakarta, the capital city of Indonesia. Between 2010 and 2016, the number of tourists visiting Pari Island grew rapidly, with Pantai Pasir Perawan being a frequently visited beach managed by a local community group^30^. On weekends, tourist numbers can reach up to 2000 people^33^.

Seagrass beds on Pari Island play a pivotal role in supporting the livelihood of local communities, particularly women who collect living shells (bivalves) for sustenance and income^35^. The dominant seagrass species found in Pari Island are *Enhalus acoroides*, *Thalassia hemprichii*, and *Cymodocea rotundata*^36,37^. Based on field measurements from 2004–2016, seagrass cover fluctuated between 164 and 271 ha^37^, with estimated aboveground carbon stocks of 200 g C m^−2^ in 2010^38^. However, estimates derived from remote sensing imagery in 2023 suggested a much lower aboveground carbon stocks of 12.4 g C m^−2^ ^39^.

In 2009, experimental seagrass restoration was initiated in Pari Island^31^. These experiments involved transplanting *Enhalus acoroides* shoots from a donor site at the northern part of the island, with survival rates ranging from 75–90% during the first three months of monitoring after planting^31^. However, monitoring was not continued due to funding limitations and the project’s discontinuation.

### Sediment sampling and analysis

Sediment cores were collected in October 2022 by manually hammering a PVC pipe (120 cm long, 6.8-cm inner diameter) into the sediment. Sampling sites were chosen based on ecosystem mapping conducted prior to fieldwork, stratified according to the distribution of four habitats: persistent meadow (seagrasses present before 2009), restored meadow (seagrass restored through transplants in 2009), bare sand (no seagrass recorded since at least 2009), and dredged (previously seagrass meadow, but excavated by dredging activities in 2017). We measured the sediment depth at core locations by gently pushing a 2-m-long stainless steel rod to the depth of refusal. We collected three sediment cores in each habitat, yielding a total of 12 cores. Sediment cores were stored vertically until opened. Compaction of sediment during coring ranged from 4 to 26%, calculated by comparing the depth of the core penetration and the depth of the sample within the core prior to core retrieval from the meadow^40^. Sediment cores were immediately sub-sectioned at 1 cm resolution for the upper 20 cm and at 5 cm resolution below 20 cm. All samples were kept frozen (− 20 °C) before analyses.

In the laboratory, all samples were dried at 60 °C until constant weight to estimate dry mass. Dry bulk density (DBD, g DW cm^−3^) was obtained by dividing the mass of the dried sample by the initial volume of the sample. Large items, such as stones and twigs, were removed, and then dry samples were ground to a fine powder using a mixer mill (MM200; Retch, Dusseldorf, Germany) and kept in an airtight container inside a desiccator.

Biogeochemical analyses were conducted at the CSIRO Environment Laboratory and the West Australian Biogeochemistry Centre in Perth, Western Australia. Sediment organic matter was estimated via loss on ignition (LOI), by combusting a subsample of ground sediment in a furnace (Thermolyne F30400, Thermo Scientific, USA) at 450 °C for 5 hours^41^. C_org_ concentration was calculated by applying the relationship between LOI and C_org_ given by Fourqurean et al.^4^.

Approximately 2 g of ground sample was acidified with HCl 4% to remove inorganic carbon, centrifuged (3400 rpm for 10 min), and the supernatant with acid residues removed carefully by pipette, avoiding resuspension of the pellet. Then, the sample was rinsed with Milli-Q water, centrifuged, and the supernatant removed. The rinsing was done twice. The pellet was re-dried and placed in a pre-weighed tin capsule for analysis of elemental composition (%C) and stable isotope ratios (*δ*^13^C and *δ*^15^N) using a continuous flow system on a Delta V Plus mass spectrometer coupled to Thermo Flush Elemental Analyser 1112 via Conflo IV (Thermo-Finnigan/Germany). Stable isotope ratios are expressed as delta (*δ*) values in parts per thousand (‰) after a normalisation procedure using international standards provided by International Atomic Energy Agency (*δ*^13^C – NBS 22, USGS 24, NBS 19, LSVEC; *δ*15N – N1, N2, USGS32) and laboratory standards^42,43^. The uncertainty of measurements was not more than 0.1‰.

The loss of mass after acidification for all the samples was more than 90%. According to Serrano et al*.*^44^, the LOI method is more accurate than elemental analysis following acidification for measuring C_org_ in seagrass sediments with high inorganic carbon. The regression analysis of the organic matter (OM or %LOI) against %C_org_ from acidification showed a weak relationship (Supplementary Figure S1). Therefore, the %C_org_ presented here was calculated from LOI. Organic carbon density (g C cm^−3^) was calculated by multiplying dry bulk density (g cm^−3^) with organic carbon concentration (%C_org_), and organic carbon stock (g C cm^−2^) was calculated by multiplying organic carbon density with sediment thickness (cm). Total sediment C_org_ stock to the bottom of the core was calculated by summing C_org_ stock for all sections from each core.

Approximately 0.3 g of ground (previously sieved through < 0.125 mm mesh) sample was also analysed for ^210^Pb to determine recent sedimentation rates. ^210^Pb was determined through the analysis of ^210^Po by alpha spectrometry after addition of ^209^Po as an internal tracer and digestion in acid media using an analytical microwave^45^. The concentrations of excess ^210^Pb to obtain the age models were determined as the difference between total ^210^Pb and ^226^Ra (supported ^210^Pb). Concentrations of ^226^Ra were determined for selected samples along each core by gamma spectrometry.

Seagrass species composition and coverage in the persistent meadows and restored areas were determined from five randomly placed 0.25 m^2^ quadrats in the vicinity of the sediment cores. Sediment depths were also recorded at each core location. Remote sensing analysis was used to estimate changes in seagrass extent at the study location. High-resolution images were downloaded from Google Earth using Google Earth Pro software at its maximum resolution. Four acquisition dates were used: December 11, 2009; September 29, 2015; August 18, 2021; and March 10, 2022. No image was available for the dredged area in 2022. The images were classified using an unsupervised method^46,47^. An ISO Cluster Classifier was used to automatically identify and cluster images into the following classes: “dense seagrass”, “seagrass” (seagrass + sand), “sand”, “mangrove”, and “hut”. The results were then converted into vector format (shapefile) and dissolved into a layer for geometric calculation to determine the area of each class in square meters.

### Statistical analyses

A Generalized Additive Model (GAM) was used to analyse patterns of %C_org_, DBD (g DW cm^−3^), and *δ*^13^C (‰) with sediment depth among habitats. To allow direct comparisons among habitats, the sediment carbon stocks (Mg C ha^−1^) were standardized to 30 cm sediment depth. Differences in carbon stocks (Mg C ha^−1^) among the four different habitats were tested using ANOVA followed by Tukey HSD. All data were examined for normality and heteroskedasticity prior to analysis. All analyses were done using the R statistical software (packages: ggplot2, tidyr, dplyr, mgcv, gam, and multcomp, R version 4.2.2^48^).

## Results

### Sediment properties

The sediment depth in the core locations ranged from 40 to 143.5 cm (Table 1). DBD along the twelve sediment profiles was on average 2.0 ± 0.4 g cm^−3^ and ranged from 0.4 to 3.4 g cm^−3^. The C_org_ concentration along sediment profiles averaged 0.98% ± 0.18% (range: 0.69% to 1.67%) and *δ*^13^C averaged − 12.31‰ ± 3.80‰ (range: − 26.65‰ to − 0.64‰).

**Table 1:** Sediment properties (mean ± SE) including sediment depth (cm), length of the samples (cm), dry bulk density (DBD), %C_org_, *δ*^13^C, accumulated carbon stock (Mg C ha^−1^) to the bottom of the core and carbon stock (Mg C ha^−1^) to 30 cm depth, from twelve cores on Pari Island.

Core ID		Sediment depth (cm)	Sample length (cm)	DBD (g cm^−3^)	C_org_ (%)	*δ*^13^C (‰)	Cstock (Mg C ha^−1^) to the bottom of the core	Cstock (Mg C ha^−1^) to 30 cm*
Persistent	N1	139	65.0	2.08 ± 0.06	1.17 ± 0.03	− 12.88 ± 0.43	125.32	76.05
	N2	132	72.5	1.80 ± 0.04	1.26 ± 0.04	− 12.31 ± 0.36	143.81	71.02
	N3	133	75.0	1.80 ± 0.06	1.16 ± 0.02	− 12.00 ± 0.61	136.64	66.28
Restored	T1	56.8	46.5	1.82 ± 0.05	0.93 ± 0.01	− 13.16 ± 0.60	70.21	55.57
	T2	49	46.5	1.78 ± 0.12	0.88 ± 0.03	− 12.91 ± 0.98	60.69	53.71
	T3	102.5	85.0	1.86 ± 0.05	0.82 ± 0.01	− 9.78 ± 0.64	122.85	50.97
Bare Sand	BS1	136	65.0	1.97 ± 0.06	0.92 ± 0.02	− 12.74 ± 0.47	111.73	50.04
	BS2	143.5	58.5	2.18 ± 0.04	0.86 ± 0.01	− 9.97 ± 0.66	95.34	58.57
	BS3	130.5	75.0	2.15 ± 0.04	0.82 ± 0.01	− 10.53 ± 0.82	115.89	51.60
Dredged	D1	139	55.0	2.39 ± 0.05	0.91 ± 0.01	− 15.23 ± 0.71	88.99	63.78
	D2	137	75.0	2.04 ± 0.04	0.97 ± 0.01	− 14.99 ± 0.26	129.85	60.05
	D3	84	50.0	2.30 ± 0.11	1.01 ± 0.02	− 10.99 ± 0.98	88.09	67.33

*We used 30 cm depth for comparison between habitats.

Results from the Generalized Additive Model (GAM) showed that DBD, %C_org_, and *δ*^13^C varied with sediment depth in each habitat (all *p* < 0.05, Table 2), and differed among habitats (persistent, restored, bare sand, and dredged, all *p* < 0.01, Table 2). However, patterns with sediment depth along the core varied among habitats for %C_org_, and *δ*^13^C (*p* < 0.01 and *p* = 0.035, respectively, Table 2). Downcore patterns in DBD did not vary significantly among habitats (*p* > 0.1, Table 2); post hoc tests revealed significant differences in DBD between restored seagrass and bare sand, as well as between restored seagrass and dredged areas (all *p* < 0.01).

**Table 2:** Results of the Generalized Additive Model at 30 cm depth to test the effect of sediment depth and habitat (Persistent (N), Restored (T), Bare Sand (BS), and Dredged (D)) on the DBD (g cm^−3^), C_org_ (%), and *δ*^13^C (‰).

Response variable	Predictor variable	F value	*p*-value
DBD	Depth	3.985	**0.047**
	Habitat	17.349	** < 0.001**
	Habitat:s(Depth)	1.838	0.1407
	T–BS		** < 0.001**
	T–D		**0.002**
%C_org_	Depth	33.864	** < 0.001**
	Habitat	149.034	** < 0.001**
	Habitat:s(Depth)	11.528	** < 0.001**
*δ*^13^C	Depth	5.260	**0.023**
	Habitat	13.467	** < 0.001**
	Habitat:s(Depth)	2.908	**0.035**

Significant effects at *p* < 0.05 are given in bold.

**Fig. 2:**
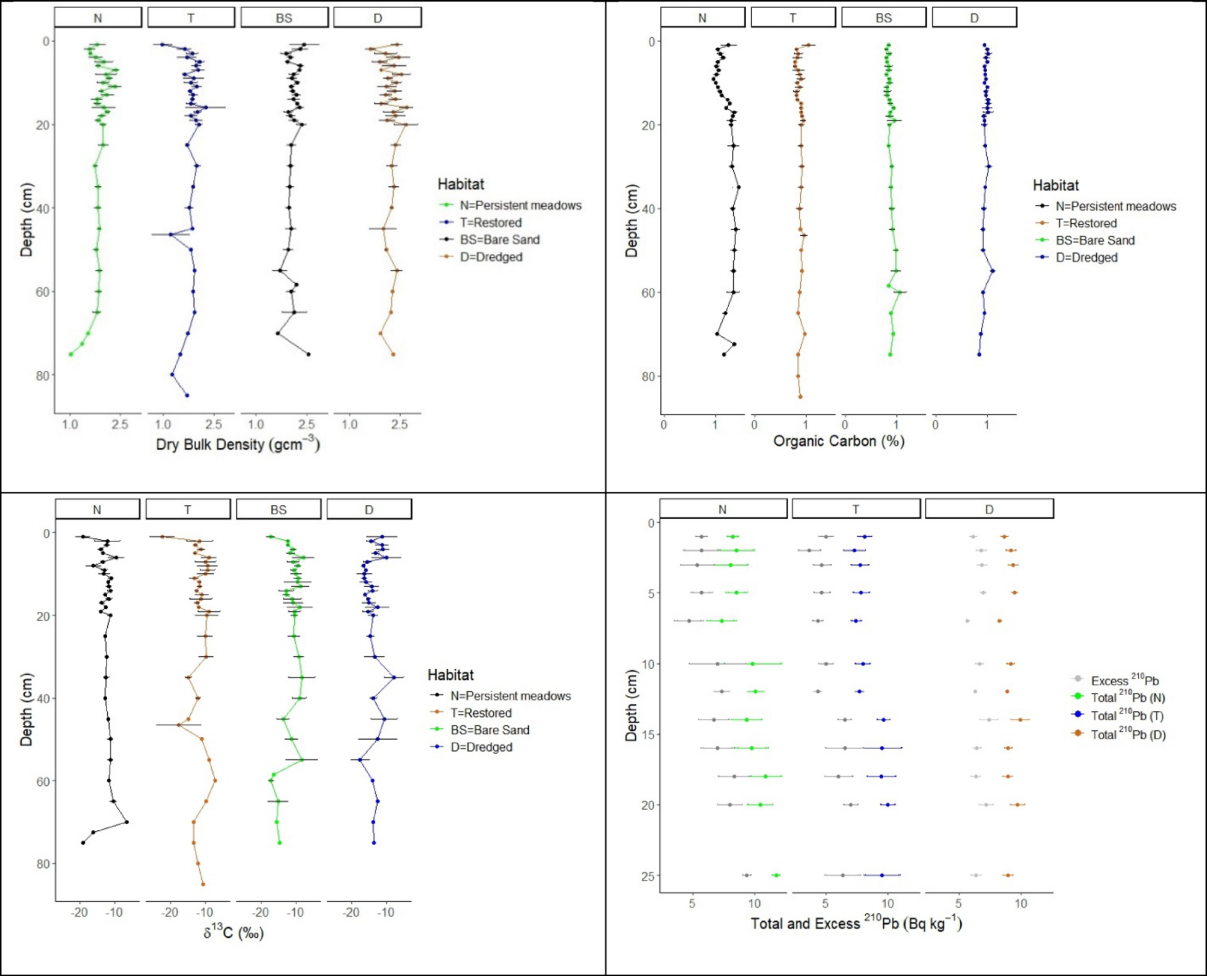
Sediment characteristics (DBD, %C_org_, δ^13^C, and Total ^210^Pb, mean ± SE) along depth profile in the sediment cores from Persistent meadows (N), Restored (T), Bare Sand (BS), and Dredged (D) areas.

The concentrations of total ^210^Pb in the sediment profiles along the upper 25 cm ranged from 6 to 14 Bq kg^−1^, 6 to 12 Bq kg^−1^, and 8 to 11 Bq kg^−1^, from the persistent meadow, restored, and dredged areas, respectively (Fig. 2). The resulting concentrations of excess ^210^Pb were extremely low for all cores and did not exhibit any trend with sediment depth. The absence of a decreasing trend in the concentrations of excess ^210^Pb with sediment depth combined with the extremely low concentrations suggest that the net sedimentation at all sites were likely negligible and indicate intense mixing of, at least, the upper 25 cm of the sediments in all habitats.

### Sedimentary organic carbon stocks at 30 cm depth

**Fig. 3:**
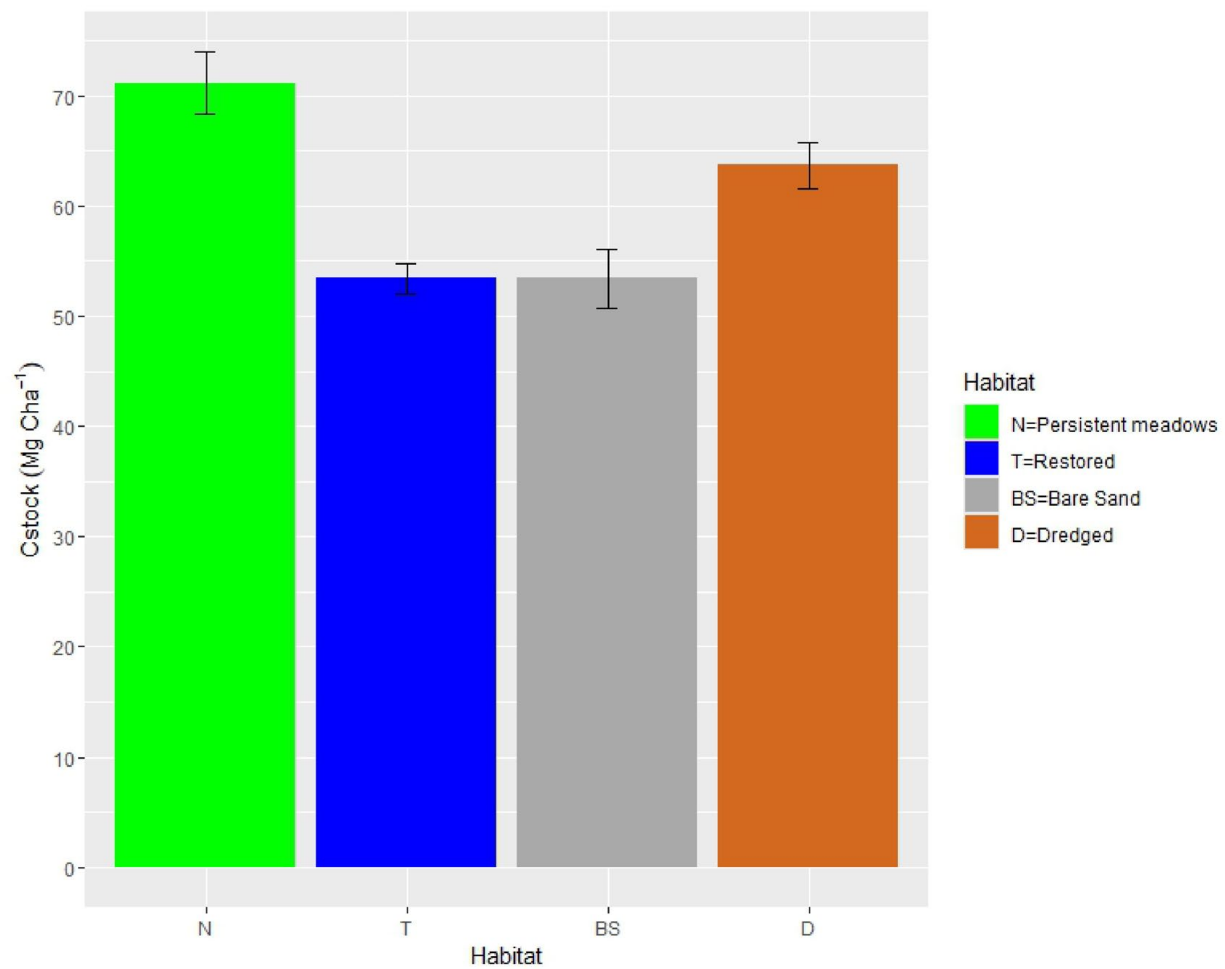
Seagrass soil carbon stocks at different habitat conditions (N = Persistent seagrass meadows, T = Restored, BS = Bare Sand, D = Dredged) in the upper 30 cm.

C_org_ stocks in the upper 30 cm (Fig. 3, Table 3) in the persistent meadows (71.12 ± 2.82 Mg C ha^−1^) were 1.3 times higher than in the bare sand and restored areas (53.4 ± 2.62 Mg C ha^−1^ and 53.42 ± 1.34 Mg C ha^−1^, respectively; F_3,8_ = 14.172, all *p* = 0.003). Meanwhile, the mean C_org_ stock in the dredged sites (63.72 ± 2.10 Mg C ha^−1^) was higher than in the bare sand and restored areas and not statistically different to persistent meadows (*p* = 0.051, and *p* = 0.182, respectively, Supplementary Table 2).

**Table 3:** DBD, %C_org_, *δ*^13^C and C_org_ stock from each core at 30 cm depth.

Core ID	DBD		C_org_		*δ*^13^C		C_org_ Stock	
	g cm^−3^	Mean ± SE	%	Mean ± SE	‰	Mean ± SE	Mg C ha^−1^	Mean ± SE
N1	2.12	1.94 ± 0.09	1.14	1.17 ± 0.02	− 13.31	− 12.81 ± 0.32	76.05	71.12 ± 2.82
N2	1.85		1.22		− 12.21		71.02	
N3	1.84		1.16		− 12.9		66.28	
T1	1.83	1.85 ± 0.02	0.93	0.87 ± 0.04	− 13.34	− 11.61 ± 1.29	55.57	53.42 ± 1.34
T2	1.83		0.87		− 12.42		53.71	
T3	1.90		0.80		− 9.08		50.97	
BS1	2.01	2.13 ± 0.06	0.89	0.84 ± 0.03	− 12.71	− 10.66 ± 1.02	50.04	53.40 ± 2.62
BS2	2.19		0.86		− 9.62		58.57	
BS3	2.18		0.78		− 9.66		51.60	
D1	2.39	2.25 ± 0.13	0.90	0.97 ± 0.04	− 14.81	− 13.90 ± 1.20	63.78	63.72 ± 2.10
D2	1.99		0.99		− 15.36		60.05	
D3	2.36		1.02		− 11.52		67.33	

**Fig. 4:**
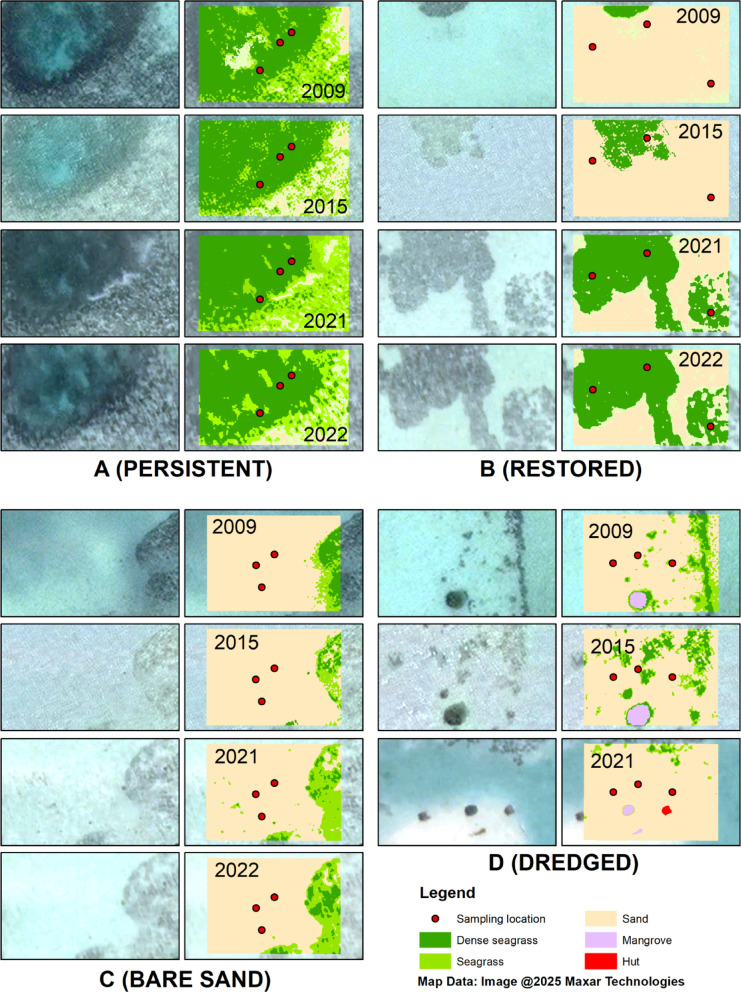
Interannual patterns in the cover of different habitats in the locations at Pari Island. A) persistent seagrass meadows, B) restored seagrass, C) bare sand, D) dredged areas. Maps were created using QGIS version 3.22.14 (Białowieża, https://qgis.org).

Remote sensing analyses (Fig. 4) revealed that dense seagrass was present at the persistent seagrass habitat continuously from 2009 to 2022. Meanwhile, in 2022, the area of restored seagrass was 22 times higher than in 2009. In the dredged area, dense seagrass decreased by approximately sevenfold from 2009 or 12-fold from 2015, as the dredging started in 2017. Bare sand areas remained mostly without seagrass, with only small patches of seagrass present outside the sampled locations in some years (Fig. 4, Supplementary Table 1).

## Discussion

At Pari Island, Indonesia, patterns in sediment organic carbon varied with depth and among habitats. Overall, sediment organic carbon concentrations (%C_org_) in persistent seagrass meadows were higher than in dredged, restored seagrass, and bare sand areas. Sediment organic carbon stocks at 30 cm depth were similar in persistent seagrass meadows and dredged areas, and higher than in restored and bare sand. Dry bulk density (DBD), %C_org_, and *δ*^13^C varied with depth but not in a consistent way across all habitats. Dredging activities at Pantai Perawan, Pari Island, led to the removal of seagrass vegetations and sediments. This study shows that the organic carbon concentrations in the dredged sediments was 17% lower than in the persistent meadows. Restoration of seagrass has resulted in large increases in cover, but no measurable increases in organic carbon concentration or stock after 13 years.

Sediment carbon stocks in tropical seagrass meadows of Pari Island were in the range of global seagrass carbon stocks of 9.21–628.1 Mg C ha^−1^ ^4^. Extrapolated to a standard 1 m depth, as in Fourqurean et al*.*^4^, the average sediment carbon stock of persistent seagrass meadows in Pari Island is 234.41 ± 8.93 Mg C ha^−1^. This estimate is higher than the conservative global estimate of 139.7 Mg C ha^−1^ ^4^ and the Southeast Asia estimate of 121.95 Mg C ha^−1^ ^49^. The organic carbon storage in Pari Island’s persistent meadows surpasses a previous estimate for Indonesian seagrass meadows of 119.5 Mg C ha^−1^ ^50^, and maximum carbon storage from published data available from South and Southeast Asia region at 205.9 Mg C ha^−1^ ^51^. This estimate is also higher than the previously published figure of 117.4 Mg C ha⁻^1^ from seagrass in Indonesian marine protected areas^52^. The average sediment carbon stocks at 30 cm depth in this study from dredged, restored, and persistent seagrasses (63.72 ± 2.10, 53.42 ± 1.34, and 71.12 ± 2.82 Mg C ha^−1^, respectively) also exceeded the current global average of 41.10 ± 1.8 Mg C ha^−1^ ^53^.

This result indicates that tropical seagrass meadows can store large quantities of organic carbon. The study helps expand knowledge about spatial variability in organic carbon of seagrass meadows across the vast extent of the Indonesian archipelago. Stankovic et al*.*^50^ reported that, up to 2022, there were 31 published studies of seagrass blue carbon in Indonesia, with 5 of this reporting sediment organic carbon. As an archipelagic country with widely varying species composition and geomorphological settings, Indonesia likely has highly variable seagrass organic carbon stock^54^. Knowing this variability helps build more accurate estimates of potential carbon stocks, which in turn helps estimate avoided emissions following conservation and enhanced carbon sequestration after restoration^51^. The high carbon stocks present in Pari Island’s seagrass meadows suggests that protection of existing seagrass should be a priority. Doing so will also maintain community livelihoods at Pari Island.

The high C_org_ stocks of the persistent seagrass meadows at Pari Island (Fig. 4A) is likely facilitated by low disturbance and persistent cover, as shown by the interannual patterns of seagrass cover. The extent of dense seagrass remained relatively constant (increasing slightly) during the period of this study. In Pari Island, *E. acoroides* is present as monospecific beds or in association with other species such as *T. hemprichii, H. uninervis, and C. serrulata*^55^. The dominance of *E. acoroides* at this location also likely influences the carbon storage capacity through its high above and below ground biomass^56^, although ^210^Pb results indicate absence of net sediment accumulation but rather that there was substantial mixing. *E. acoroides*, with ribbon-like leaves that can grow to one meter or more tall^55^, tends to facilitate fine sediment deposition due to its deeper, larger and more persistent rhizomes, which preserve more refractory carbon than labile forms of carbon^57^.

The loss of seagrass can result in loss of sediment C_org_ accumulated over decades and even centuries, as observed in other studies where coastal development and mooring activity led to erosion and loss of C_org_ stock^15,58^. At Pari Island, we found that sediment in dredged areas had 17% lower %C_org_ than persistent seagrass meadows in the upper 30 cm. We also expected to find lower C_org_ stocks in dredged areas, but DBD was higher, and thus the C_org_ stock in the upper 30 cm was still comparable to that of persistent meadows. High DBD might be due to low sediment porosity in the area, which in some studies showed negative relationships with %C_org_ concentrations^59,60^. The dredged areas show distinct δ^13^C in the top 5 cm of sediment compared to the persistent seagrass (Fig. 2), potentially indicating different sources of carbon in this area. In depth investigation is needed to draw stronger conclusions about the sources of carbon in the sediment.

We had hypothesised elevated C_org_ concentrations in surface sediment within restored seagrass meadows, which would indicate return of carbon sequestration. However, this study showed that there was no significant increase of C_org_ concentration in restored meadow 13 years after seagrass planting. DBD, %C_org_, and *δ*^13^C varied with depth, and the downcore patterns and Generalized Additive Model (GAM) results also showed inconsistent patterns, making it difficult to draw strong conclusions. These are likely due to the intense mixing of the sediment, as evidenced from the ^210^Pb concentration profiles, so it might take a long period before accumulation of organic carbon is detectable. Similar slow recovery of carbon accumulation and sequestration under restored seagrasses was reported from the temperate seagrass *Posidonia australis* in Shark Bay^61^, in south-western Australia^24^, and restored *Zostera marina* in Bohai sea^62^.

Organic carbon in the sediment is entrained by the seagrass canopy^63^. However, if seagrass density is insufficient, this effect will be less pronounced and deposited sediment can undergo greater resuspension compared to dense seagrass meadow^64^. This is reflected from the seagrass coverage in the restored area at Pari Island, which averages 66.33%, compared to 85% coverage in persistent meadows (Supplementary Table 3). Nevertheless, the visible expansion of seagrass in restored areas is promising for restoration as a viable method to accelerate recovery of degraded seagrass meadows and this encourages further research and efforts to support these initiatives.

Carbon accumulation rates following restoration might take many years before they are comparable to undisturbed seagrass meadows^24,25,61^. Various studies show different intervals for increases in sediment organic carbon after restoration: 12 years in Virginia, USA^25^, and 18 years in south-western Australia^24^. Similarly, in mangrove ecosystems, the return of carbon storage capacity after restoration activities varies: 17 years in Melbourne, Australia^65^, between 15 and 40 years to sequester the equivalent of biomass carbon stock lost^66^. Despite the expansion of seagrasses in restored areas on Pari Island (Fig. 4), the C_org_ concentration and stocks remain similar to those in bare sand. Approximately 13 years after planting, neither C_org_ nor total stocks in restored meadows are comparable to persistent seagrass in any metrics, possibly due to the smaller coverage in the restored areas.

Carbon sequestration functions can be recovered through seagrass restoration, but this process is likely to be slow, as demonstrated by this study and others. Understanding local environmental conditions, including the causes of impact and hydrodynamics, is essential for effective seagrass management and restoration planning^16,67,68^. If generating carbon credits is one of the goals for seagrass restoration, returns are likely to take a long time, and there should not be an expectation of an immediate return. A comprehensive assessment of greenhouse gas (GHG) accounting in Virginia, USA^69^, showed that net carbon sequestration benefits may become apparent after 12 to 15 years, but the potential carbon offset credits were considered marginal relative to the high costs of restoration. The study also recommended considering other seagrass ecosystem benefits, alongside carbon sequestration, such as coastal protection and fisheries support, to enhance incentives for restoration. An initial assessment is recommended to confirm that the restoration site meets the requirements for demonstrating organic carbon accumulation^70^. Additionally, a new approach using Geographic Information System (GIS)-based tools or using Habitat Suitability Modelling (HSM) may be applied to identify suitable areas for restoration^70,71^, supported by the availability of adequate and high-resolution data.

## Conclusion

This study demonstrates the capacity of persistent seagrass meadows to store organic carbon in Pari Island, Indonesia. Sediment in dredged areas had lower C_org_ concentration than sediment in persistent meadows. Restored seagrasses showed meadow expansion, but no enhanced organic carbon accumulation was detected in the sediment after 13 years. Therefore, promoting the conservation and maintenance of well-preserved meadows is crucial, especially because coastal infrastructure is projected to increase in many countries^72^. Awareness of the emissions resulting from the removal of seagrass meadows should be raised, because restoring seagrass after disturbance demands considerable time and resources to return carbon sequestration rates to their natural levels.

## Electronic supplementary material

Below is the link to the electronic supplementary material.

Supplementary Material 1

## Data Availability

The datasets generated during and/or analysed during the current study are available from the corresponding author on reasonable request.
