# Supplementary material for: Impacts of dredging and restoration on sedimentary carbon stocks in seagrass meadows of Pari Island, Indonesia
Source: Sci Rep. 2025 Jul 15;15:25551. doi: 10.1038/s41598-025-03870-3 (PMC12264270; doi:10.1038/s41598-025-03870-3)
Supplement: Supplementary file 1 — Supplementary Material 1 [file 41598_2025_3870_MOESM1_ESM.docx]

**Supplementary**


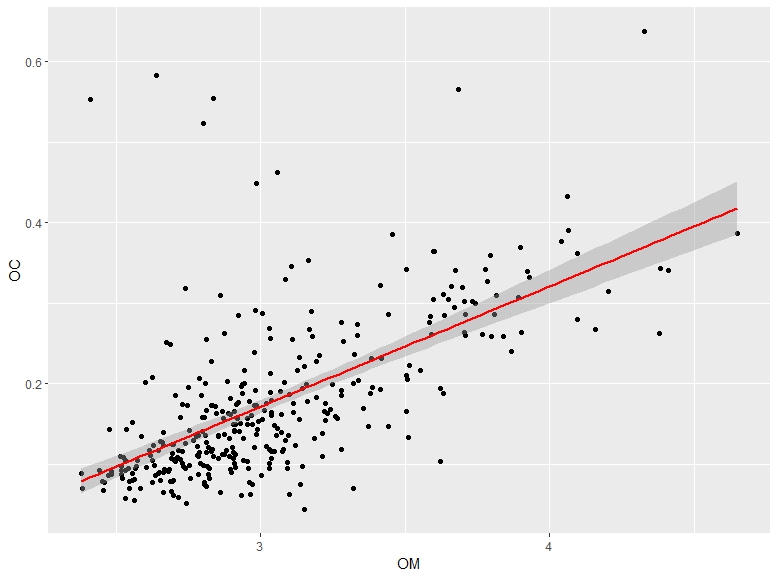


y = 0.1492x - 0.2758

R² = 0.3935

Figure S1. Regression analysis from ratio of organic carbon (measured by Elemental Analyser) and organic matter (% LOI) from all cores in Pari Island.

Supplementary Table 1. Changes in areas using remote sensing analyses.

| Habitat | Classification | Area (m^2^) | | | |
| --- | --- | --- | --- | --- | --- |
|  |  | 2009 | 2015 | 2021 | 2022 |
| N | Dense seagrass | 1533.71 | 1551.14 | 1382.00 | 1554.40 |
|  | Seagrass | 754.52 | 512.25 | 984.99 | 631.66 |
|  | Sand | 279.11 | 503.95 | 200.35 | 381.28 |
|  | Total | 2567.34 | 2567.34 | 2567.34 | 2567.34 |
| T | Dense seagrass | 80.37 | 454.87 | 1689.30 | 1789.43 |
|  | Seagrass | 9.16 | 0.88 | - | - |
|  | Sand | 3144.63 | 2778.41 | 1544.86 | 1444.73 |
|  | Total | 3234.16 | 3234.16 | 3234.16 | 3234.16 |
| BS | Dense seagrass | 158.12 | 65.35 | 68.98 | 142.25 |
|  | Seagrass | 152.33 | 135.12 | 366.72 | 344.77 |
|  | Sand | 2045.13 | 2155.11 | 1919.88 | 1868.56 |
|  | Total | 2355.58 | 2355.58 | 2355.58 | 2355.58 |
| D | Dense seagrass | 124.85 | 196.44 | 16.14 | NA |
|  | Seagrass | 239.16 | 199.29 | 55.84 | NA |
|  | Sand | 1543.38 | 1488.52 | 1841.42 | NA |
|  | Mangrove | 33.90 | 57.04 | 17.89 | NA |
|  | Hut | - | - | 10.00 | NA |
|  | Total | 1941.29 | 1941.29 | 1941.29 | NA |

Supplementary Table 2. ANOVA results for C_org_ stock at 30 cm depth.

| Parameter | F value | P value |
| --- | --- | --- |
| C_org_ stock | 14.1720 | 0.001* |
| T-BS |  | 1.000 |
| D-BS |  | 0.051 |
| N-BS |  | 0.003* |
| D-T |  | 0.052 |
| N-T |  | 0.003* |
| N-D |  | 0.182 |

Supplementary Table 3. Seagrass cover at persistent meadows (N) and restored areas (T), Ea=*Enhalus acoroides,* Th=*Thalassia hemprichii*.

| Core ID | Seagrass species | Seagrass cover (averaged from 5 quadrats) |
| --- | --- | --- |
| N1 | Ea | 71% |
| N2 | Ea, Th, Cr | 89% |
| N3 | Ea, Th, Cr | 96% |
| Average N | | 85.33% |
| T1 | Ea, Th | 76% |
| T2 | Ea, Th | 68% |
| T3 | Ea, Th | 55% |
| Average T | | 66.33% |
